# A machine vision platform for measuring imbibition of maize kernels: quantification of genetic effects and correlations with germination

**DOI:** 10.1186/s13007-018-0383-7

**Published:** 2018-12-21

**Authors:** Nathan D. Miller, Scott C. Stelpflug, Shawn M. Kaeppler, Edgar P. Spalding

**Affiliations:** 1 Department of Botany, University of Wisconsin, 430 Lincoln Drive, Madison, WI 53706 USA; 2 Department of Agronomy, University of Wisconsin, 1575 Linden Drive, Madison, WI 53706 USA

**Keywords:** Germination, High-throughput phenotyping, Imbibition, Image analysis, Machine vision, *Zea mays*

## Abstract

**Background:**

Imbibition (uptake of water by a dry seed) initiates the germination process. An automated method for quantifying imbibition would enable research on the genetic elements that influence the underlying hydraulic and biochemical processes. In the case of crop research, a high throughput imbibition assay could be used to investigate seed quality topics or to improve yield by selecting varieties with superior germination characteristics.

**Results:**

An electronic force transducer measured imbibition of single maize kernels with very high resolution but low throughput. An image analysis method was devised to achieve high throughput and sufficient resolution. A transparent fixture held 90 maize kernels in contact with water on the imaging window of a flatbed document scanner that produced an image of the kernels automatically every 10 min for 22 h. Custom image analysis software measured the area *A* of each indexed kernel in each image to produce imbibition time courses. The ultimate change in area (ΔA) ranged from 19.3 to 23.4% in a population of 72 hybrids derived from 9 inbred parents. Kernel area as a function of time was fit well by $A\left( t \right) = A_{f} \left( {1 - e^{ - kt} } \right)$ where *A*_*f*_ is the final kernel area. The swelling coefficient, *k*, ranged from 0.098 to 0.159 h^−1^ across the genotypes. The full diallel structure of the population enabled maternal genotype effects to be assessed. In a separate experiment, measurements of kernels of the same 25 inbreds produced in three different years demonstrated that production and storage variables affected imbibition much less than genotype. In a third experiment, measurements of 30 diverse inbred lines showed that *k* varied inversely with germination time (r = − 0.7) and directly with germination percentage (r = 0.7).

**Conclusions:**

Nonspecialized imaging hardware and custom analysis software running on public cyber infrastructure form a low-cost platform for measuring seed imbibition with high resolution and throughput. We measured imbibition of thousands of kernels to determine that genotype influenced imbibition of maize kernels much more than seed production and storage environments. In some hybrids, *k* depended on which inbred parent was maternal. Quantitative relationships between *k* and germination traits were discovered.

**Electronic supplementary material:**

The online version of this article (10.1186/s13007-018-0383-7) contains supplementary material, which is available to authorized users.

## Background

The water potential of a mature seed can be as low as − 100 MPa [1, 2], while that of moist soil may be close to 0 MPa. A seed sown in that situation inevitably imbibes water from the soil down the water potential gradient (Δψ). The rate of imbibition depends on the magnitude of Δψ and the ease with which water can flow across the outer seed coverings at soil contact points [3, 4]. The imbibed water causes the seed to swell and gain weight. Gravimetric and volumetric methods have been devised to measure imbibition time courses. The methods entail removing seeds from an imbibition scenario to determine either their weight or their displacement volume, then returning them to imbibe more, and repeating the measurement over a series of time points [5, 6]. Imbibition time courses thus obtained typically display a rapid initial phase followed by a gradual slowing as Δψ approaches zero many hours later [3]. Shull [7] may have been the first to recognize the exponential character of the swelling curves obtained for *Xanthium* seeds. An ensuing period of no net water uptake can persist for many hours, days, or months depending on whether other preconditions for germination have been met. In the case of minimal dormancy, hydration of the embryo directly activates metabolism and cell expansion and the plateau period is short. Whether after a long or short plateau period, post-imbibition water uptake coupled to cell expansion pushes the radicle tip through the seed coat to achieve germination [8].

Imbibition, as a prerequisite for germination, is an essential first step in establishing a new seedling or an entire crop. It is a critical and vulnerable phase in a plant’s life cycle. Rapid imbibition can reduce structural integrity of seeds, allowing cellular materials to leach out and pathogens to enter. This is a particularly serious issue for crop stand establishment when soil temperatures are low [9]. At low temperatures, metabolism may not be active enough to repair or combat the consequences of physical damage due to rapid swelling resulting in reduced seedling survival rates [10].

Despite the importance of imbibition in the establishment of a crop, current methods cannot treat the process as a trait for breeding or a subject of mechanistic investigations because existing methods for measuring it are imprecise and low throughput. Researchers cannot scale up traditional manual gravimetric or volumetric methods to characterize genetically structured populations or the effects of multiple treatments in any reasonable scenario because a person must handle each sample at each time point to perform a measurement. An automated method for measuring imbibition could enable investigations with a larger scope than is currently feasible. For example, automation would enable study of the effects on imbibition of many different conditions, genotypes, and combinations of conditions and genotypes in order to understand the controlling genetic factors and how environmental effects interact with them. The results could help explain variation in stand establishment, which is the percentage of seeds that give rise to a viable seedling. Rapid and synchronous establishment of the stand can significantly impact the ultimate yield [11, 12].

The research effort reported here used maize kernels to develop an automated, large-scale method for precise measurements of imbibition time courses. Maize kernels are caryopsis-type fruits bounded by a pericarp rather than an anatomically less complicated testa (seed coat), but this difference does not affect the principles of imbibition. Maize kernels were found to increase in volume by 41% over 24 h of imbibition [5]. The pericarp of a maize kernel is a diploid maternal structure formed by fusion of the ovary wall and the integuments of the ovule. The triploid endosperm and the diploid embryo within the kernel have maternal and paternal genomes. Variation in maize kernel imbibition could therefore be due to the genetic constitution of the maternal parent, both parents, and non-genetic environmental effects, which conceptually could include the maternal environment during development, and environmental influences after the fruit is separated from the mother plant. The method we developed to measure imbibition time courses was tested in experiments designed to investigate the genetic and non-genetic sources of variation in maize kernel imbibition.

## Results

### Imbibition force measurement and analysis

**Fig. 1:**
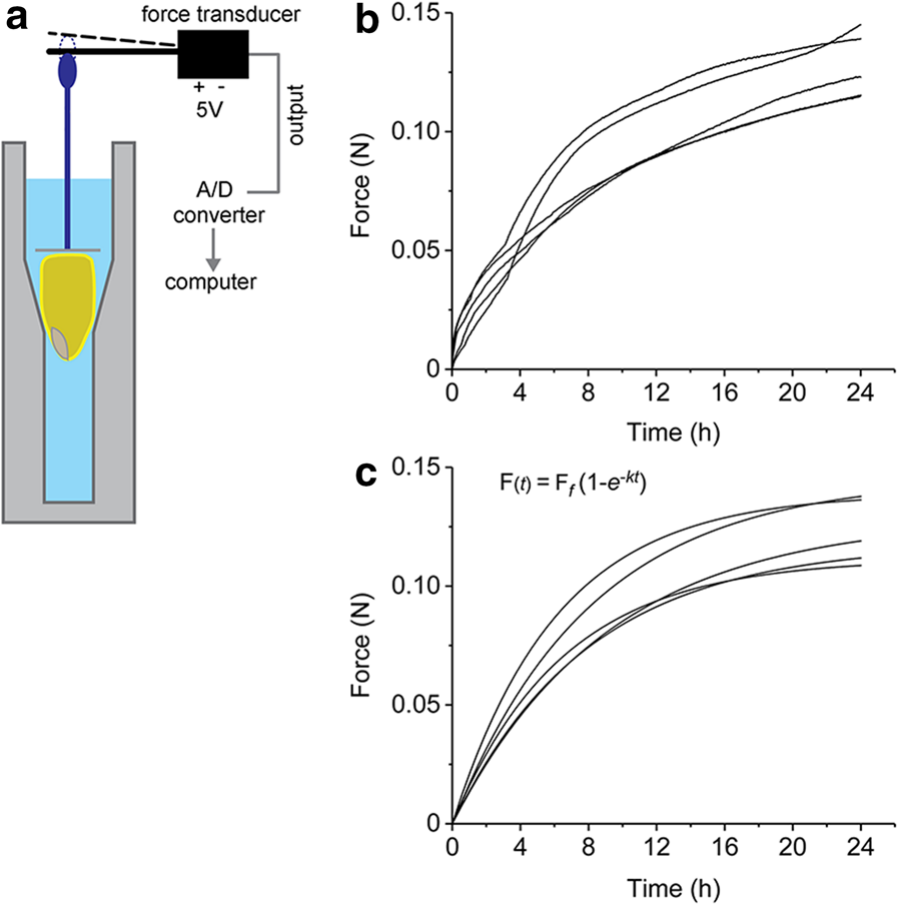
High resolution measurement of kernel swelling during imbibition with an electronic force transducer. **a** Diagram of an apparatus consisting of spectrophotometer cuvette to hold a kernel in contact with water and a plastic rod that pushes on the leaf of a force transducer as the kernel swells. The analog voltage output of the transducer is linearly related to force. An analog to digital (A/D) converter digitizes the output and a computer records the result every second. **b** Five example traces obtained with Mo17 inbred kernels. **c** Curves fit to the five examples in **b**

One approach to measuring imbibition is to determine the mass of a seed sample at time points following contact with water. Another is to measure the volume of liquid a sample of seeds displaces in a graduated cylinder. Repeating the measurement of mass or displaced volume at time intervals can produce a swelling curve, which can be analyzed to determine the rate and extent of the imbibition process. To improve on the resolution of these manual methods, we arranged an electronic force transducer to contact the surface of a maize kernel held in a chamber that could be filled with water (Fig. 1a). The transducer produced a voltage directly proportional to the force exerted by the kernel as it swelled. Recording the voltage output at 1-s intervals for 24 h produced high-resolution imbibition time courses shown in Fig. 1b. Kernels of a standard inbred genotype of field corn (Mo17) were used in this pilot study. The force time course curves were fit well by a single rising exponential function, $F\left( t \right) = F_{f} \left( {1 - e^{ - kt} } \right)$ where $F_{f}$ is the final force and *k* is the constant that characterizes the rate of change, what Leopold [5] called the swelling coefficient. The average *k* determined from the best fits of the equation to the Mo17 curves shown in Fig. 1c was 0.13 ± 0.01 h^−1^. By comparison, Leopold [5] used a volumetric method to find *k* = 0.157 h^−1^ for the Merit sweet corn hybrid.

These results demonstrated that a force transducer can measure imbibition with very high time resolution. The force transducer method could be useful when mechanistic questions require precise measurements of the initial rates of water entry. However, the throughput of the method is low. To treat imbibition rate as a phenotype to be measured in large populations of maize lines, or in any type of screening study, a high throughput method is required. To address the need for high throughput, an image-based method was developed.

### Image acquisition and analysis

**Fig. 2:**
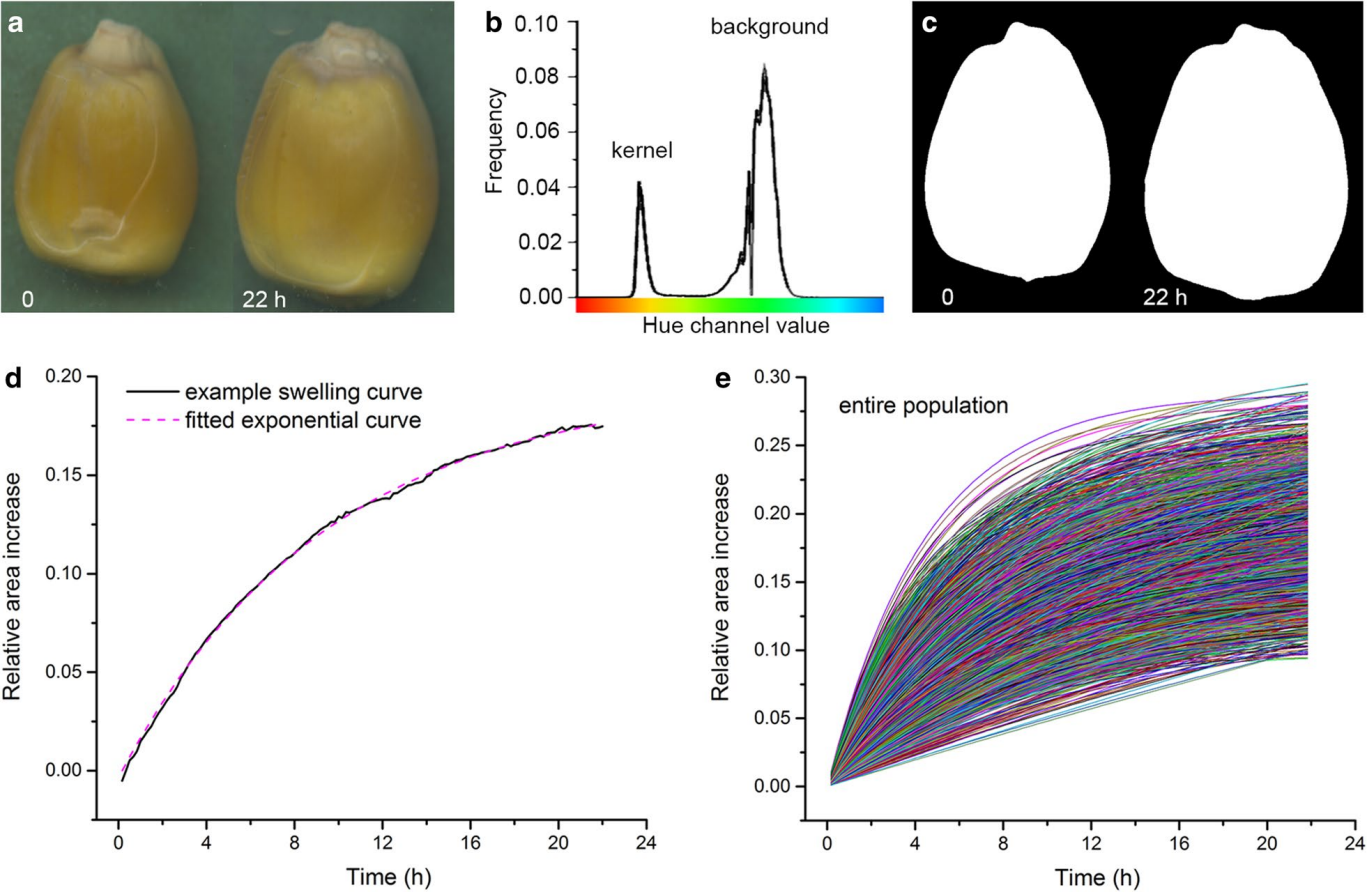
Summary of the image-based method for measuring imbibition and the data it produces. **a** Image of a kernel at the beginning and end of an imbibition trial (22 h). **b** Frequency histogram of the hue channel value in a three-color image of a kernel against the wet paper background. **c** Binary version of the image in **a** produced by applying a threshold to the histogram exemplified in **b**. **d** Example of a swelling curve produced by determining kernel area (**a**) at each 10-min time point, expressed as a fraction of the initial area, and a fit of the indicated equation to the raw data (dashed line). **e** Approximately 5000 fitted imbibition curves show the range of variation observed in this study

The image-based method relies on one or more previously described [13] flatbed document scanners capable of capturing images at 1200 dots per inch at 10-min intervals for 22 h. A bank of six such scanners operating in parallel to increase throughput produced the data presented here. Custom shell scripts executing on a computer running a Linux operating system controlled each scanner to achieve the required automation. Kernels were equally spaced in 10 rows and 9 columns on the surface of agar held in a square, transparent, plastic dish. The dish remained on the bed of the scanner during the 22 h recording period to create a time lapse recording of the 90 kernels. The resulting 0.2 gigabyte images were automatically stored as 8-bit red/green/blue (RGB) Tag Image Format (TIF) files. A kernel cropped from images acquired at 0 and 22 h is shown in Fig. 2a.

The area of each kernel was extracted from the collected images by the following custom-coded algorithm. First, the full RGB image was transformed to the hue/saturation/value (HSV) color space. The two distinct peaks in the hue channel histogram (Fig. 2b) correspond to yellowish kernels and the water-saturated cyan paper laid over the kernels. A full-image mask *M* was created by setting all pixels with a hue value less than 0.2 or greater than 0.8 to background status. This removed the edges of the plastic dish and other components outside of the scene but retained the pixels comprising kernels and the surrounding cyan space in the foreground. Next, sub-images within *M* containing single kernels were defined by crop boxes centered at points determined by calculating the standard deviation of hue values in a window slid along the horizontal and vertical directions within *M*. Local peaks in the standard deviation were taken as the row and column coordinates for the centers of the crop boxes, which were set to 500 × 500 pixels. This method for producing sub-images corresponding to individual kernels relies on each kernel being placed precisely in rows and columns. Because kernels sometimes moved during assembly of the seed-containing fixture, an adjustment routine re-centered each crop box at the center of mass of the largest binary object within the initial crop box, which was always a kernel. A threshold step [14] applied to the hue value histogram corresponding to each of the sub-images produced by the adjusted crop box produced a binary object corresponding to the kernel. Remaining holes in the object were filled with a close operation to produce a mask of the sub-image as shown in Fig. 2c. Area (*A*) was defined as the number of pixels in a kernel mask. A fully functional instance of the code is deployed as a Web service in the Discovery Environment at CyVerse along with example image stacks to demonstrate the function. A potential user should visit https://de.cyverse.org/de/, set up an account, and ask the authors to grant access to the tool.

*A* was measured for each kernel in each image of the automatically-collected time series to form imbibition curves as shown in Fig. 2d. The equation $A\left( t \right) = A_{f} \left( {1 - e^{ - kt} } \right)$ in which *A*_*f*_ is the final area, fit the imbibition curves very well, as was also found by Leopold [5]. The value of *k* for this individual kernel was 0.017 h^−1^, consistent with the values obtained by the force transducer method. The remainder of this study equates the values of *k* with the swelling coefficient, the initial area *A*_*i*_ as a measure of dry kernel size, and the normalized difference between *A*_*f*_ and *A*_*i*_, or ΔA, as the percentage increase in kernel size resulting from imbibition. Figure 2e shows the curves fit to each of more than 5000 imbibition data sets generated during this study.

### Effects of kernel source on imbibition

**Fig. 3:**
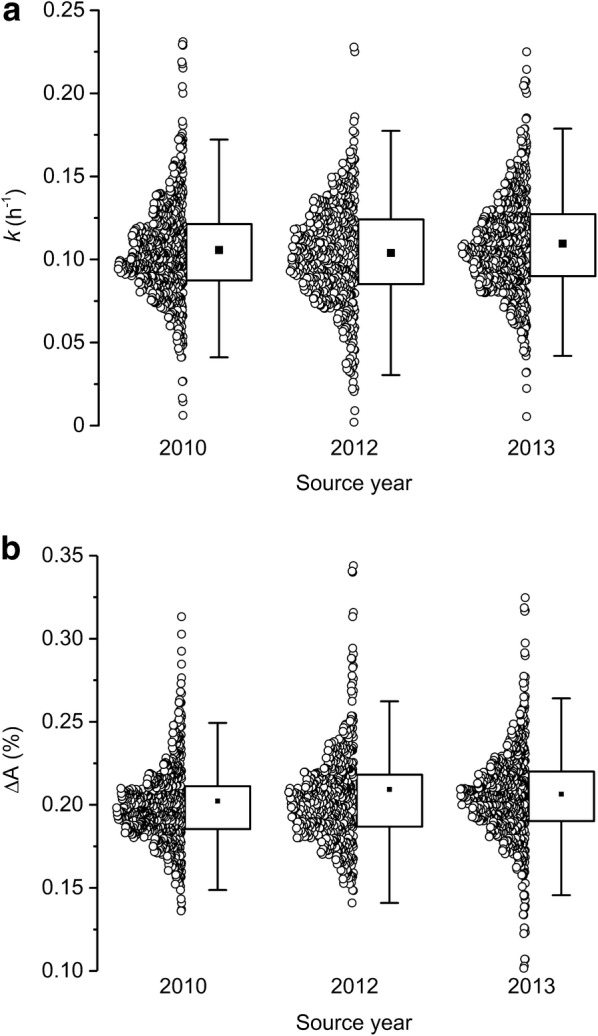
Distributions, means, and quartiles of **a** swelling coefficients, *k* and **b** final percent change in kernel area, ΔA for 27 kernels of the same 25 genotypes produced in 3 different years and stored until measured

We obtained kernels of 25 maize inbred genotypes (lines) produced during the summers of 2010, 2012, and 2013 (Additional file 1) to determine the extent to which source year and storage variables would affect the results relative to genetic factors. In each of 3 trials for each of 3 source years, each genotype was represented by 9 kernels (one row). Thus, the experiment required 2025 separate imbibition measurements. Each imbibition time course produced a value for *k* and for ΔA. These values for each kernel source year are plotted in Fig. 3. The distributions of *k* (Fig. 3a) and ΔA (Fig. 3b) values for each source were very similar. We then conducted an analysis of variance to quantify effects attributable to kernel source, genetic factors, and any technical biases in the platform. We found a component of variation linked to the column in which the kernel was placed in the sample holder. This small but significant effect was probably due to a systematic ‘fisheye’ lens effect near the periphery of the scanner bed (Table 1). The column effect reduced *k* and made the kernels appear larger only in columns 1 and 9. A small decrease in *k* was linked to trial number (1, 2, or 3), perhaps due to biased selection of kernels to assay first from a limited pool. Critically, the analysis of variance showed that the genotype effect on *k* was 3.6-fold greater than the kernel source effect (Table 1). Genotype also exerted the largest effect on the ΔA trait (Table 1). Thus, the image-based method is suitable for genetic mapping and breeding studies of imbibition, the first stage in the establishment of a maize crop.

**Table 1:** Mean squares (MS) and F values, fixed-effects model of imbibition traits from three seed sources

Sources of variance	df	MS *k*	MS ΔA	F value *k*	F value ΔA
Genotype	24	0.0157	0.0093	43.8***	15.0***
Seed Source	2	0.00036	0.0038	12.2***	6.1**
Column	8	0.00078	0.0053	6.6***	8.6***
Replication	2	0.00028	0.016	9.6***	25.2***
Residuals	1897	0.0282	0.00062	–	–

Genotypes represent a random sample of 25 inbreds from the WiDiv diversity panel, listed in Additional file 1: Table 1. Seed sources represent inbred seed stocks produced in summer nurseries spanning 2010, 2012, and 2013. The analysis of variance was performed on the mean phenotypic values of 9 kernels per replication after omitting outliers with studentized residuals that exceeded ± 3. Residuals represent the interaction of all terms included in the model

*k*, swelling coefficient; ΔA % increase in kernel area after 22 h; df, degrees of freedom

***p <0.001; **p <0.01

### Full-diallel analysis to assess combining ability and maternal effects

**Fig. 4:**
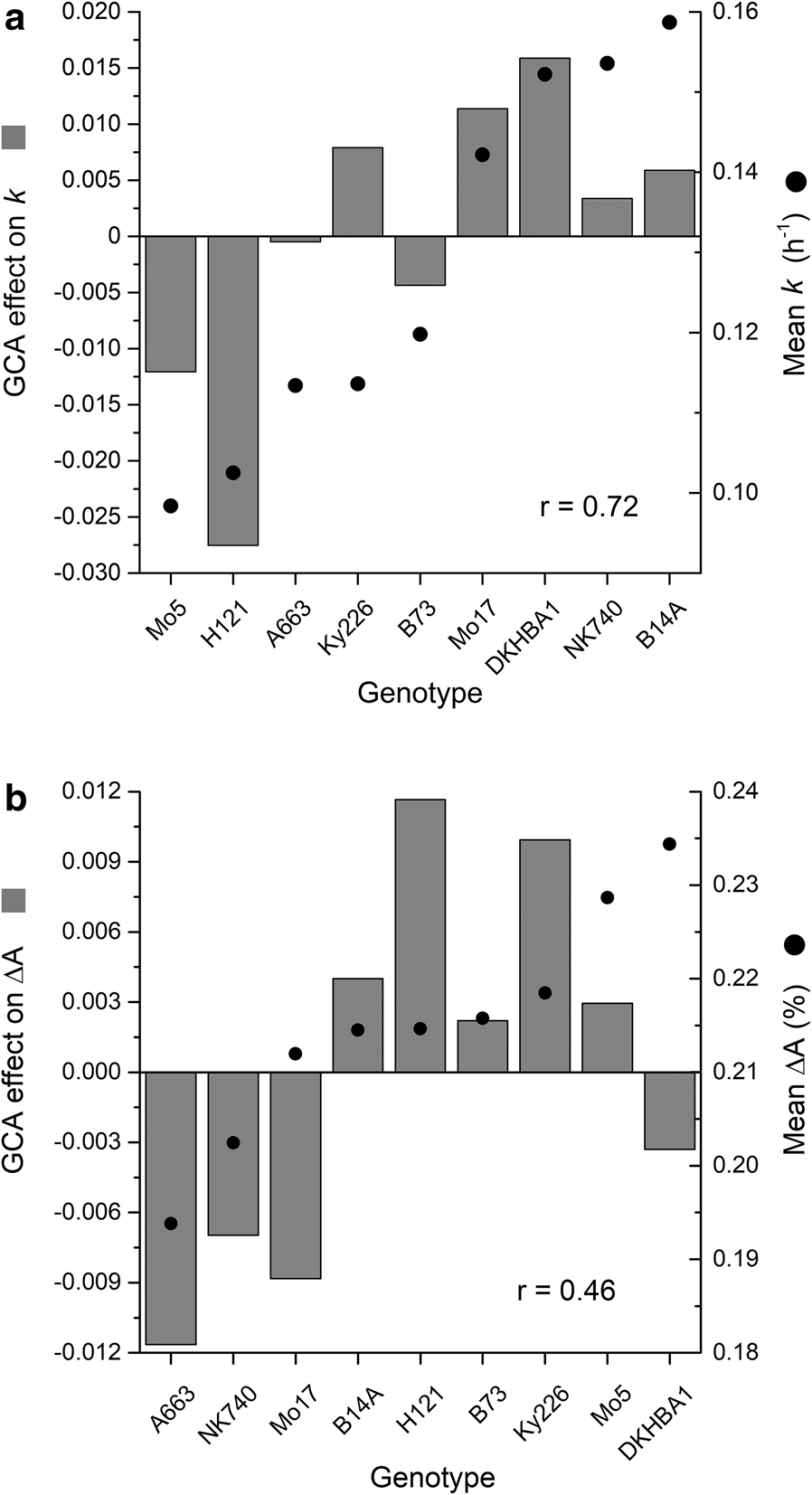
General combining ability (GCA) as evidence for genetic control of the **a** *k* and **b** ΔA imbibition traits in the diallel parental genotypes. Co-plotting the means of the traits for each genotype in ascending order illustrates the relationship with GCA, quantified by a Pearson’s correlation score

We used the imaging and analysis platform to quantify imbibition of kernels representing a population of 72 hybrids constructed from 9 inbreds crossed in a full-diallel structure. Results obtained with the hybrid kernels were subjected to analysis of variance to determine general combining ability (GCA). The GCA main effects, defined as the average performance of a parent among all crosses [15], were determined for each parent (Fig. 4a). The large GCA effects with respect to *k* indicated a strong additive genetic contribution to this trait within this set of lines (Table 2). Based on GCA effects, the best inbred parents for producing hybrid kernels with faster imbibition were DKHBA1, Mo17, and Ky226. Because the GCA effect on the *k* trait was correlated with the mean value of *k* (r = 0.7), crossing any two high-*k* inbred parents may be expected to produce a fast-swelling hybrid. The same analysis was conducted on the swelling capacity (ΔA) trait. The best inbreds for producing hybrid kernels with the highest swelling magnitude were H121, Mo5 and Ky226. Figure 4b shows that the correlation between the GCA effect on ΔA and the mean for the trait was 0.58, less than for *k*.

**Table 2:** Combining abilities and reciprocal effects of imbibition traits

Estimates of quadratic components	*k*	ΔA
GCA (*φ*_*g*_)	4.9	5.7 × 10^−5^
SCA (φ_s_)	8.1	9.2 × 10^−5^
RE (φ_*re*_)	9.5	1.5 × 10^−4^
2φg/2φg + φs	0.55	0.56

Quadratic components associated with the fixed-effects of general combining ability (GCA), specific combining ability (SCA), and reciprocal effects (RE) for the swelling constant (*k*), and change in area (ΔA) from the full-diallel experiment. The ratio 2φg/2φg + φs assessed the relative importance of general and specific combining abilities in determining progeny performance

**Fig. 5:**
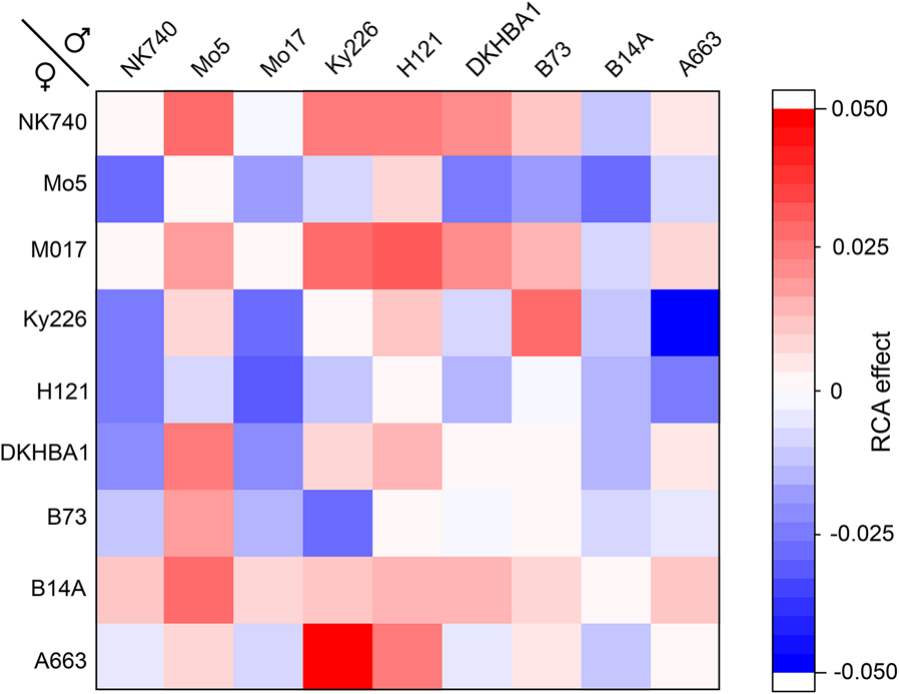
Reciprocal combining ability (RCA) effects on the swelling coefficient *k*. The results indicate which genotypes contribute differently to *k* of a hybrid kernel when the inbred serves as the mother instead of the father. The RCA effect is zero if *k* of an A × B hybrid equals *k* of a B × A hybrid

Because the pericarp is considered to be a major barrier to water uptake during imbibition, and because other maternally-derived kernel components could be influential, a hybrid kernel may imbibe differently depending on the cross construction. The full diallel structure of this experiment permitted such reciprocal combining ability (RCA) effects to be assessed (Fig. 5). The crosses A663♀ × Ky226♂, Mo17♀ × H121♂, and NK740♀ × Mo5♂ showed the highest positive RCA effects for *k* (Fig. 5). The closely related NK740 and Mo17 lines [16] generally contributed positive RCA effects on *k* when used as females, which may be counter-intuitive given that these genotypes belong to the non-stiff stalk maize heterotic group typically used as males during seed production. Therefore, Mo17 and NK740 would serve well as females if the goal was to speed up imbibition in a hybrid. A663 is an example of an inbred that generally displays minimal RCA effects except when it is the female in a cross with Ky226. In this case, it has a positive RCA effect on the swelling coefficient. The significant effects on *k* imparted by mother plants could be considered when selecting parents to produce hybrids with desired germination characteristics.

### Correlation between imbibition traits

We compared *k* and ΔA measured in the diallel and kernel source experiments. The mean and standard deviation of *k* was 0.11 ± 0.03 h^−1^ for the kernel source experiment and 0.13 ± 0.18 h^−1^ for the diallel experiment. Mean ΔA was 20.2% ± 0.03 for the kernel source experiment and 21.1% ± 0.01 for the diallel. Pearson’s correlation analysis performed on the results of both experiments indicated that *k* and ΔA were negatively correlated (*p* < 0.001) for the kernel source (*r* = − 0.259, *n *= 1896) and diallel experiments (− 0.434, *n *= 2916). This result indicates that faster swelling kernels ultimately swell to a lesser degree than slower-swelling kernels. The swelling coefficient exhibited a minor, but significantly positive correlation to initial seed area in the diallel experiment (*r *= 0.104, *p *< 0.001) but was not significantly correlated in the seed source experiment (*r *= − 0.015).

### Relationships between imbibition rate and germination

**Fig. 6:**
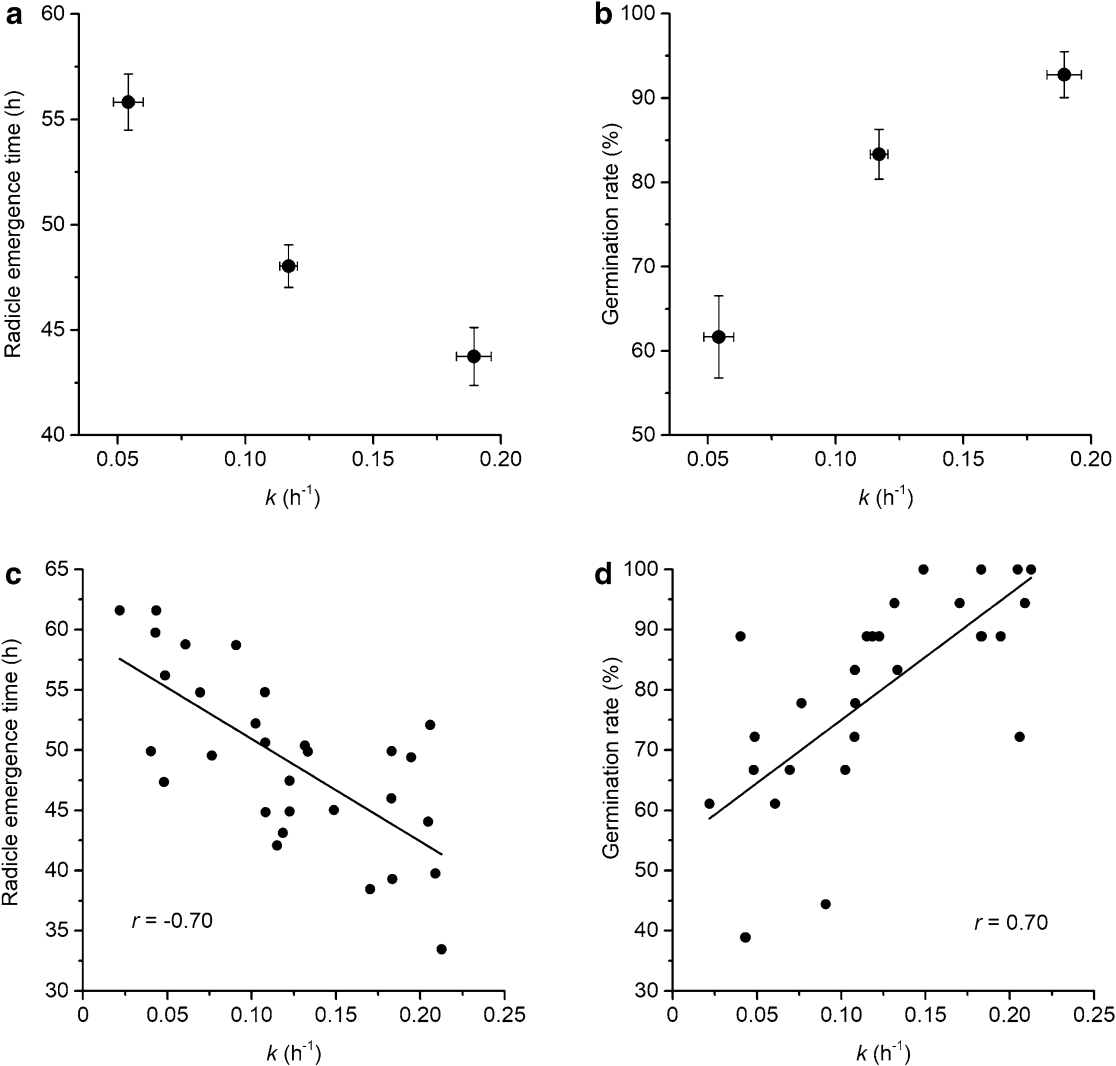
Relationship between imbibition and germination traits. The results are based on three sets of 10 inbred lines selected from a population of 500 for displaying either low, medium, or high swelling coefficients (*k* values). **a** The average time between sowing and radicle emergence for the low, medium, and high *k* sets. **b** The percentage of kernels that ultimately germinated (germination rate) for the low, medium, and high *k* sets. Pooling the results of all 30 genotypes showed how *k* correlated with **c** radicle emergence time and **d** germination rate, defined as the percentage of kernels that ultimately germinated

We investigated the relationship between *k* and the germination process that occurs approximately 2 days later. First, we screened 500 inbred maize lines from the WiDiv population and then selected 3 sets of 10 that displayed high, medium, or low *k* values. The selected genotypes are listed in Additional file 2. Average *k* values of the 30 genotypes ranged from 0.21 h^−1^ (genotype CI90C) to 0.022 h^−1^ (genotype Il14H). Figure 6 shows that the mean *k* of the ten genotypes in the low set was 0.054 ± 0.02 h^−1^. It was 0.12 ± 0.01 h^−1^ for the medium group and 0.19 ± 0.02 h^−1^ for the high set. Kernels from the three sets were sown and repeatedly scanned in the same agar-based fixtures used to measure the imbibition parameters. Inspection of the image series determined the radicle emergence time for each kernel that successfully germinated. The mean radicle emergence time for the low *k* set was 55.8 h, which was significantly different than 48.0 h for the medium set, which was significantly different from 43.7 h for the high set (Fig. 6a). The 3 *k* sets also differed significantly with respect to the percentage of kernels that ultimately germinated (germination rate). The mean for the low set was 61.6%, which was significantly different from 83.3% for the medium set, which was significantly different from 92.7% for the high *k* set (Fig. 6b). Combining the three sets in one scatterplot (Fig. 6c) showed that *k* and time to radicle emergence were strongly negatively correlated (Pearson’s *r* = − 0.70, *p* = 1.60 × 10^−5^). Figure 6d shows that *k* and germination rate were significantly positively correlated (Pearson’s *r* = 0.70, p = 1.66 × 10^−5^) within these 30 genotypes selected to cover the range of *k* values in the WiDiv population. The results in Fig. 6 indicate that time to germination and final percent germination are reasonably predictable given an hours-long automated imbibition assay based on computational image analysis.

## Discussion

Mapping quantitative trait loci (QTL) or otherwise investigating associations between genotype and phenotype requires large sample sizes, especially when the goal is to identify superior variants in breeding project. The new measurement capability reported here creates an opportunity to treat imbibition as a phenotype for such large-scale genetic experiments because the throughput of the assay makes it technically feasible to collect sufficient data. In addition to appropriate throughput, which derives from the degree of automation in the acquisition and analysis, the platform produces phenotype data that are accurate and reliable. The mean *k* for the Mo17 genotype determined by a force measurement was 0.134 ± 0.010 (n = 5) and the completely independent image analysis approach determined *k* to be 0.127 ± 0.003 (n = 18). These results were not significantly different. The method also proved to be capable of detecting substantial variation due to genetics against a background of non-genetic variation (Tables 1 and 2, Figs. 4 and 5). Plant breeders require accurate measurement of the genetic component of variation in a trait in order to selectively modify it. Therefore, this phenotyping platform could be useful in breeding efforts to alter the imbibition characteristics of maize.

Once a population possessing sufficient genetic and phenotypic variation has been measured, fitting an equation to the imbibition data will produce the ΔA and *k* parameters that summarize the time course. These single values can be the phenotypes to be mapped for the purpose of identifying genetic elements that control them. Follow-up work can elucidate molecular details about the mechanisms controlling imbibition. However, it is not necessary to reduce the time course data to parameters of a preordained equation in order to perform genetic mapping. Statistical methods have been developed that enable the entire data set to be treated as a time course without specifying a fixed mathematical model of the curve. Plant phenotype data collected by automated image analysis of root gravitropism provided the inspiration for some of these new methods [17–19]. The results make it possible to learn when during a response a particular region of the genome began, and ceased, to contribute to a dynamic phenotype. Imbibition phenotype data sets may be well suited for analysis by some of these functional methods. Because the equation $y\left( t \right) = A_{f} \left( {1 - e^{ - kt} } \right)$ fits many of the imbibition responses well, it may seem unnecessary to do more than map the ΔA and *k* parameters. However, close inspection of the curves indicates that there can be structure in the time courses that deviates from the exponential. Some responses appear to show evidence of a biphasic process that would not be captured by fitting any simple equation to the results, but which could be captured by so-called functional approaches to QTL analysis. Regardless of how this dynamic seed phenotype is modeled for statistical genetic analyses, the platform described here creates the essential quantitative description.

The relationship between the swelling coefficient and subsequent germination (Fig. 6) indicates that plant breeders could use the platform to select for germination characteristics by measuring imbibition as a correlated proxy trait. Another potential application of the platform is to assess the quality of a seed lot. Many aspects of establishing a vigorous and synchronous crop depend on the quality of the sown seeds, including germination potential. Traditional methods for assessing germination potential of a seed lot use wet paper towels, several days of incubation, and then visual inspection of the outcome by a trained technician [20]. The results presented here raise the possibility of an hours-long automated assay producing results that to some extent, perhaps a large extent, predict the outcome of the traditional method.

Essentially pure water (water potential of near 0 MPa) was in close contact with the kernels throughout the imbibition process in all of the experiments reported here. This high external ψ maximizes the Δψ on which imbibition directly depends [3, 8], though it may be far from the expected condition in many natural and agricultural environments. The interaction between water availability and genotype during imbibition is an example of an important topic in seed biology that the platform presented here is well-suited to address. The agar bed and overlaid wet paper could be prepared with an osmoticum instead of pure water to reduce the external water potential to a specific negative value. Using the platform to determine how a mapping population of kernels swells could identify genetic loci that contribute proportionally more to imbibition when water availability is restricted than when it is maximum. Such a beneficial interaction between genotype and environment in maize could be improved upon through a breeding program enough to have a practical effect on crop performance.

Basic seed biology research could also benefit from the platform. A large variety of mutations in maize affect kernel morphology and anatomy [21, 22]. The effects range from subtle to profound. Precisely determining the effects of mutations on the swelling time courses could, especially in combination with magnetic resonance micro-imaging [23], help determine which internal seed structures limit or affect the rate of imbibition. For example, kernels with one (wild type) or multiple aleurone layers [24] could be compared to determine if this outermost living tissue affects swelling as much as pericarp thickness, which is also genetically controlled [25]. Kernels with different types and amounts of starch, or amounts of free sugars could be measured and compared to learn how these chemical traits affect imbibition. With perhaps minor modifications, the method is expected to perform well on seeds of other species.

## Conclusions

The high-throughput and precise method for measuring seed swelling described and demonstrated here enables researchers to treat imbibition as a phenotype in statistical genetic studies. The platform could also be used to measure effects of many conditions or treatments in addition to identifying controlling genetic elements. Inexpensive, standard scanners acquire the images; the analysis tool is available as a Web service on public computing infrastructure. Thus, the technical barrier to performing large scale studies of an important process in the life cycle of a plant has been significantly lowered.

## Materials and methods

### Imbibition force measurement

An electronic force transducer (FORT 10 g, World Precision Instruments) excited with ± 5 V produced by a BK Precision DC power supply was used to measure swelling of a single kernel held in a semi-micro acrylic spectrophotometer cuvette (Fig. 1a). A thin plastic cap on top of the kernel formed a flat surface to push a plastic rod with a conical tip up against the measuring leaf of the transducer after the addition of distilled water caused the kernel to swell (Fig. 1). The unfiltered output of the transducer, proportional to applied force, was digitized at a frequency of 1 Hz for 24 h after addition of water by a computer equipped with an A/D converter and custom software written in the LABVIEW (National Instruments) programming language. The 86,400 point time series was smoothed by the Sovitzky-Golay method with a window of 500 points using Origin (Microcal) analysis software to produce the traces shown in Fig. 1b. The transducer output increased linearly with known test masses used to convert output voltage to force units.

### Image acquisition

The method requires seeds to be uniformly in contact with water and accessible to imaging optics. We accomplished this with a system based on square plastic bioassay dishes (Corning #43111, 245 mm × 245 mm) containing 150 mL of 1% agar. Nine kernels per genotype were placed one inch apart to form a row. Ten such rows spaced one inch apart filled a dish with a grid of 90 kernels on an agar bed. The kernels were covered with one layer of green paper (8.5 in. × 11 in., Avery Paper Company) to provide background color contrast. Two layers of paper towel, evenly soaked with 20 mL of deionized H_2_O, were placed on top of this paper. Each assembled dish was placed on a flatbed scanner (Epson Perfection V700 photo flatbed scanner) for image acquisition. Imbibition proceeded at room temperature (20 °C).

### Plant material

Kernels of 25 maize inbred lines produced during the summers of 2010, 2012, and 2013 were used to investigate how different production environments and storage durations affected imbibition parameters. The genotypes of the 25 inbred lines are presented in Additional file 1. In order to determine how a genotype contributes to imbibition when combined with another genotype, a full diallel population of 72 hybrids was studied. The population was created by intercrossing 9 parents, using each parent once as the male, and once as the female. The parent genotypes were A663, B14A, B73, DKHBA1, Ky226, H121, Mo5, Mo17, NK740. The kernels were produced during the summer of 2014 at the West Madison Agricultural Research Station. One seed source for each hybrid was used in the experiment. Kernels from a diverse set of inbred lines within the Wisconsin Diversity Panel [abbreviated WiDiv; [16, 26] were used to test correlations between imbibition traits and germination rates.

### Experimental design and statistical analysis

Analysis of variance of general combining ability (GCA), specific combining ability (SCA), and reciprocal combining ability (RCA or reciprocal effects) of the full diallel were calculated using the *diallel1* function based on Griffing’s diallel model 1 [27], contained within the *plantbreeding* package of R software [28]. Parents and replicates are considered fixed in this analysis because they are specifically chosen inbred lines.

The 81 genotypes (72 reciprocal hybrids, 9 inbred parents) were evaluated in an experiment comprised of four replicates blocked in time with subsamples of nine seeds representing a genotype within a replicate. After filtering of outlier values with studentized residuals > |3|, mean values of the nine kernels were calculated and used for analysis of each replication. A genetic model that includes both additive and dominance effects [27] was used:

$$y_{\text{ijl}} =\upmu + g_{\text{i}} + g_{\text{j}} + s_{\text{ij}} + r_{\text{ij}} + b_{\text{l}} + e_{\text{ijl}} ,$$

where *y*_ij_ = the mean trait value of the hybrid F_1_s and reciprocals (*i*, *j* = 1, 2,…p); µ is the general population mean; *g*_i_, *g*_j_ = the effect of general combining ability (GCA) effect for the *i*th and *j*th parents, respectively; *s*_ij_  = the effects of specific combining ability (SCA) for crosses between the *i*th and *j*th parents such that *s*_ij_ = *s*_ji_; *r*_*ij*_ = the reciprocal effect (RE) that measures the differences provided by the parent of *i* or *j* when used as a male or female in cross *ij*, such that *r*_ij_ = − *r*_ji_, *b*_l_ is the effect of replicate l = 1…4; e_ijkl_ = the average experimental error/residual effects associated with the *ijl*th individual observation. Significance of GCA, SCA, and RE effect ≠ zero was tested via *F*-test as $F = GCA^{2} /var\left( {\widehat{{g_{i} }}} \right)$, $SCA^{2} /var\left( {\widehat{{s_{ii} }}} \right)$ or $SCA^{2} /var\left( {\widehat{{s_{ij} }}} \right)$), and $RE^{2} /var\left( {\widehat{{r_{ij} }}} \right)$, with degrees of freedom equal to the number of observations minus 1.

### Evaluating relationships among seed imbibition and germination traits

In order to determine if the speed of imbibition was related to speed of germination, we screened 500 inbred lines from the WiDiv population [16, 26]. Based on *k* values, we selected the ten fastest, the ten slowest, and ten intermediate swelling-rate genotypes. Total germination percentage and radical emergence time was measured in these 30 genotypes. The germination assay was performed with the same apparatus and planting method as the imbibition assay, except that images were acquired every 30 min for 72 h and each plate contained only 45 kernels to allow space for root growth. A trial consisted of nine kernels per genotype. Two blocked trials were performed. Radicle emergence time was scored by inspecting the image sequences, not automatically by image processing. Germination rate for a genotype was the number of kernels with emerged radicles after 72 h divided by the total number of kernels present.

## Additional files

**Additional file 1.** Information about inbred lines used for the seed source and diallel experiments. Table of genotypes, pedigrees, year of kernel production

**Additional file 2.** Genotypes selected to evaluate the relationship between *k* and germination. Table of genotype, swelling category, swelling coefficient, and germination metrics
