# Supplementary material for: A machine vision platform for measuring imbibition of maize kernels: quantification of genetic effects and correlations with germination
Source: Plant Methods. 2018 Dec 21;14:115. doi: 10.1186/s13007-018-0383-7 (PMC6302439; doi:10.1186/s13007-018-0383-7)
Supplement: Supplementary file 1 — Additional file 1. Information about inbred lines used for the seed source and diallel experiments. Table of genotypes, pedigrees, year of kernel production [file 13007_2018_383_MOESM1_ESM.pdf]

**Additional file 1**

**Pedigree, heterotic groupings, release year and origin information of inbred lines used for the seed source experiment and those used as parents of the full-diallel population.** Yellow colored cells indicate inbred lines used in both the full-diallel and seed source experiments. SSS: stiff-stalk synthetic; NSS: non-stiff stalk; POP: popcorn; PVP: Plant Variety Protection lines; LH: Holden's

| **Genotype** | **Pedigree** | **Heterotic Group** | **Year** | **Origin** | **PVP** |
|---|---|---|---|---|---|
| A663 | A427(3)X Cuzco | unclassified | 1975 | U of Minnesota | non |
| B14A | Cuzco X B14 (8) | SSS | 1962 | Iowa | non |
| B73 | BSSS | SSS | 1972 | Iowa | non |
| CH157 | NA | unclassified | . | Ontario, Canada | non |
| DKHBA1 | PH3195 X PH3199 | unclassified | 1986 | United States | PVP-Dekalb |
| H121 | Syn. HCBSA | unclassified | . | Purdue | non |
| H99 | Illinois Syn. 60C | NSS | 1974 | Indiana | non |
| IDS69 | NA | POP | . | Iowa | non |
| Ky226* | NCIaDDC X Coah. 8 | unclassified | 1966 | Kentucky | non |
| LH145 | A632 X CM105 | SSS | 1984 | Iowa | PVP-LH |
| Mo17 | C.I.187-2 X C103 | NSS | 1966 | Missouri | non |
| Mo5 | K55(3) X N6 | unclassified | 1958 | Missouri | non |
| MoG | Mastadon | unclassified | . | . | non |
| NC230 | K55 X Yellow line or hybrid | unclassified | . | North Carolina | non |
| ND262 | NDSF | unclassified | . | N Dakota | non |
| NK740 | Mexican_Deep_Kernel X Mo17(4) | unclassified | 1988 | Minnesota | PVP-NTP |
| Oh43 | W8 X Oh40B | NSS | 1949 | Ohio | non |
| PH207 | PHG3BD2 X PHG3RZ1 | NSS (Iodent) | 1984 | Iowa | PVP-Pioneer |
| PHB47 | SD105 X B37(3) | SSS | 1984 | Iowa | PVP-Pioneer |
| PHW65 | PH861 X PH595 | NSS | 1988 | Iowa | PVP-Pioneer |
| PHZ51 | PH814 X PH848 | NSS | 1986 | Iowa | PVP-Pioneer |
| SD40 | P3709 | unclassified | . | S Dakota | non |
| Tr | Reid Yellow Dent (Troyer strain) | unclassified | 1938 | Purdue | non |
| Va99 | Oh07B X Pa91 | unclassified | . | Virginia | non |
| W22 | Ill_B10 X W25 | NSS | 1946 | Wisconsin | non |
| WU-TAN-TZAO | NA | unclassified | . | Shaanxi, China | non |

***** indicates the line was used only in the full-diallel experiment and not seed source
