# Supplementary material for: A machine vision platform for measuring imbibition of maize kernels: quantification of genetic effects and correlations with germination
Source: Plant Methods. 2018 Dec 21;14:115. doi: 10.1186/s13007-018-0383-7 (PMC6302439; doi:10.1186/s13007-018-0383-7)
Supplement: Supplementary file 2 — Additional file 2. Genotypes selected to evaluate the relationship between k and germination. Table of genotype, swelling category, swelling coefficient, and germination metrics [file 13007_2018_383_MOESM2_ESM.pdf]

Additional file 2. Genotypes selected from a survey of 500 members of the WiDiv association panel to evaluate the potential relationship between swelling coefficient *k* and germination characteristics. The ten fastest, ten slowest, and ten intermediate imbibing genotypes from the WiDiv panel were subjected to germination assays. *k*, swelling coefficient; ΔA, % change in kernel area; RET, radicle emergence time; GR, germination rate.

Genotype	Group	*k* (h^{-1})	ΔA (%)	RET (h)	GR (%)
WIL900	fast	0.195	22.6	49.4	88.9
PHT22	fast	0.183	21.9	46.0	88.9
CI90C	fast	0.213	23.5	33.5	100.0
Ki43	fast	0.183	22.5	39.3	88.9
CML323	fast	0.209	23.4	39.8	94.4
N200	fast	0.205	16.9	44.1	100.0
NC338	fast	0.183	20.7	49.9	100.0
CML322	fast	0.149	19.4	45.0	100.0
WIL500	fast	0.206	18.6	52.1	72.2
NC356	fast	0.170	21.1	38.5	94.4
W64A	medium	0.108	18.0	44.9	77.8
CML218	medium	0.122	19.4	47.5	88.9
PHG50	medium	0.102	16.6	52.2	66.7
SG30A	medium	0.132	17.7	50.4	94.4
B109	medium	0.122	16.5	44.9	88.9
H124w	medium	0.115	19.1	42.1	88.9
SD15	medium	0.108	18.4	50.6	83.3
NC326	medium	0.118	19.2	43.1	88.9
LH82	medium	0.133	16.6	49.9	83.3
L139	medium	0.108	15.5	54.8	72.2
Il101T	slow	0.043	38.9	61.6	38.9
W7151	slow	0.049	30.2	56.2	72.2
P39	slow	0.043	35.6	59.8	38.9
CL17	slow	0.061	20.8	58.8	61.1
Il14H	slow	0.022	60.0	61.6	61.1
CL18	slow	0.040	37.4	49.9	88.9
A641	slow	0.076	20.8	49.6	77.8
Yong28	slow	0.091	20.6	58.7	44.4
C42	slow	0.048	32.9	47.4	66.7
EP1	slow	0.069	17.3	54.8	66.7
